# Modeling of Vaccination and Contact Tracing as Tools to Control the COVID-19 Outbreak in Spain

**DOI:** 10.3390/vaccines9040386

**Published:** 2021-04-14

**Authors:** Mª Àngels Colomer, Antoni Margalida, Francesc Alòs, Pilar Oliva-Vidal, Anna Vilella, Lorenzo Fraile

**Affiliations:** 1 Department of Mathematics, ETSEA, University of Lleida, E-25198 Lleida, Spain; colomer@matematica.udl.cat (M.À.C.); pilaroliva@ca.udl.cat (P.O.-V.); 2 Department of Game Resources and Wildlife Management, Institute for Game and Wildlife Research, IREC (CSIC-UCLM-JCCM), E-13005 Ciudad Real, Spain; 3 Primary Health Center, Passeig Sant Joan, 08010 Barcelona, Spain; fralos.bcn.ics@gencat.cat; 4 Public Health Department Hospital Clínic de Barcelona, 08036 Barcelona, Spain; avilella@clinic.cat; 5 Department of Animal Science, ETSEA, University of Lleida, 25198 Lleida, Spain; lorenzo.fraile@udl.cat; 6 Department of Animal Science, Agrotecnio, University of Lleida, 25198 Lleida, Spain

**Keywords:** control measures, vaccination, population dynamic P system, Spain

## Abstract

We developed an agent-based stochastic model, based on P Systems methodology, to decipher the effects of vaccination and contact tracing on the control of COVID-19 outbreak at population level under different control measures (social distancing, mask wearing and hand hygiene) and epidemiological scenarios. Our findings suggest that without the application of protection social measures, 56.1% of the Spanish population would contract the disease with a mortality of 0.4%. Assuming that 20% of the population was protected by vaccination by the end of the summer of 2021, it would be expected that 45% of the population would contract the disease and 0.3% of the population would die. However, both of these percentages are significantly lower when social measures were adopted, being the best results when social measures are in place and 40% of contacts traced. Our model shows that if 40% of the population can be vaccinated, even without social control measures, the percentage of people who die or recover from infection would fall from 0.41% and 56.1% to 0.16% and 33.5%, respectively compared with an unvaccinated population. When social control measures were applied in concert with vaccination the percentage of people who die or recover from infection diminishes until 0.10% and 14.5%, after vaccinating 40% of the population. Vaccination alone can be crucial in controlling this disease, but it is necessary to vaccinate a significant part of the population and to back this up with social control measures.

## 1. Introduction

A novel coronavirus (SARS-COV-2) spread quickly in China at the end of 2019, and then rapidly in other areas. This new coronavirus causes a disease named COVID-19 whose clinical expression varies widely between patients from an asymptomatic infection to a severe disease requiring treatment in a hospital critical care unit. Many different symptoms have been associated with this disease, varying from patient to patient. The most common symptoms are cough, fever, shortness of breath or difficulty breathing, fatigue, muscle or body aches, headache, loss of taste or smell, sore throat, and diarrhea [1,2,3,4,5]. A percentage of patients eventually die as a consequence of the disease. Mortality rates vary by country as well as by other factors including patient age, ethnicity, underlying diseases/co-morbidities, and medical treatments applied [6].

The various waves of this viral pandemic have been difficult to control globally and resulted in considerable fear within the populations affected as well as severe economic impacts as governments and health authorities have struggled to devise effective control measures [7,8,9,10]. At the beginning of the outbreak, the control of the pandemic in Europe was mainly based on strict social measures including social distancing and population lockdowns [11,12,13]. While these have proved effective in the short term, they have high economic costs as well as other consequences such as widespread mental health issues, with unforeseeable impacts on society over the long run [14,15,16]. Social measures, based on social distancing, strict hand hygiene, and the wearing of face masks have been proposed as key measures to control this pandemic [17]. In some countries, it is compulsory to always wear a face mask as a key measure to limit virus transmission and decrease the reproduction ratio (R number) of the disease in the population [18,19].

Vaccination programs focused on limiting the transmission of infectious agents within a population have provided considerable human health benefits [20,21]. Vaccination reduces the susceptibility of an individual to infection as well as the chances of an infected individual passing the infection to others. If enough people in a population achieve this reduced susceptibility and infectivity, ‘herd immunity’ is said to have been achieved and this certainly benefits a population greatly [22,23]. The chances of an individual becoming infected decrease as the proportion of people vaccinated in a population rises, similar to the situation in livestock diseases [24]. Pharmaceutical companies have made huge efforts to develop vaccines against SARS-COV-2 to control the disease at the population level in the shortest period of time. Many different approaches to developing efficacious vaccines against SARS-COV-2 are in various stages of development [25] and currently three vaccines are available and approved for use in the USA and Europe [26,27].

Mathematical models can provide new insights into the epidemiology of infectious diseases and suggest criteria for the design of more efficient control strategies. Population dynamic P system (PDP) models (inspired by the biology of cell function) provide an alternative to traditional modeling for the study of complex problems [28]. They allow handling of a very large number of interactions in a more efficient way [29,30] and have not previously been applied in human epidemiology. A stochastic model based on PDP modeling has been developed to study the course of COVID-19 epidemics under several different epidemiological scenarios. This model [31] currently allows studying pandemic dynamics in different interrelated geographical areas, and the effects of different control measures in reducing the spread of the disease, e.g., polymerase chain reaction (PCR) tests carried out by health personnel; vaccines; and administrative measures such as limiting the number of people present at hospitality venues and meetings, the closure of communal activities, the establishment of regional lockdowns, and other precautionary measures.

This paper sets out to disentangle the effects of vaccination and contact tracing to control COVID-19 outbreak at population level where control measures (social distancing, mask wearing and hand hygiene) are or not applied.

## 2. Materials and Methods

### 2.1. PDP COVID-19 Model

The PDP COVID-19 model described here is a PDP based stochastic model [31], which allows the study of the course of a contagious disease outbreak under different epidemiological scenarios. The complete model is included in the Supplementary Material (Figures S1–S3, for details see [31]). The scenarios are described by values for the parameters of the model (Tables S1 and S2) that are usually inherent to the disease itself (e.g., probabilities of disease transmission, incubation time, and contagious period) and by the measures applied in order to control it (e.g., population movement restrictions, wholesale population testing, diagnosis, and vaccination). The time unit in the PDP COVID-19 model is one day. The outcomes of the simulation are the number of people who died or who recovered from infection (symptomatic plus asymptomatic). The sum of these two variables estimates the number of infected people. 

Our goals were to study and compare the effects of two control measures: vaccination and contact tracing using diagnostic tests, with and without social control measures in place. The social control measures considered are social distancing, hand hygiene, and consistent face mask wearing outside of the home. As previously published by our research group, the probability of transmission of this virus, from infected to non-infected people, is close to 10% if the virus is in free circulation [31]. Protection of 100% of the population by vaccination will, at least in the short term, be difficult to achieve. This study models a variety of more realistic scenarios, whereby 10%, 20%, 30% and 40% of the population are vaccinated over the near future (during the year 2021). Those vaccinated were chosen at random from the model population. While there are several different diagnostic tests, we focused on the subsequent contact tracing and PCR test for all the contacts traced. Moreover, all the persons who have had close contact with a COVID-positive person (contact tracing) are identified and required to self-quarantine. Since it is difficult, if not impossible, to trace 100% of the contacts, the effects on the course of the pandemic of tracing 10%, 20%, 30% and 40% of the contacts was studied. Because social control measures are simple and can be applied simultaneously with other measures, such as contact tracing and population vaccination, we ran two sets of models for the percentages of vaccination and or diagnostic tests/contact tracing, with and without social control measures.

### 2.2. Statistical Study and Sensitivity Analysis

All statistical analyses were performed using the R Core package [32] (R Core 2018, https://www.R-project.org/) (accessed on 15 September 2020). The COVID-19 PDP model was used to execute two Box–Behnken designs, each with three factors at two levels and two response variables. The response surface gives the mathematical relationship between the independent variables and the response variables, using a quadratic model. This surface was used to study the sensitivity of the PDP model to the values of the statistically significant independent variables. The population modeled had no restrictions on normal activities (work, schools, the use of restaurants and people movements). Each design involved 16 sets of experiments. The factors in the first design were: the number of people infected at time 0 (10,000 or 50,000); the percentage of the population protected by vaccination (10% or 40%); and the probability of disease transmission from infected to non-infected people (0.05 or 0.10). The factors in the second design were the same, except for the percentage of people protected by vaccination (assuming non transmission after vaccination), which altered according to the percentage of contacts with positives traced (0% or 40%). The outcomes of the simulations were the number of people who died and who recovered from infection (both symptomatic and asymptomatic). Four response surfaces were obtained, one for each dependent variable. 

### 2.3. Ethical Statement

Not necessary because of the entirely theoretical approach of this study.

## 3. Results

### 3.1. The Effects of Vaccination 

#### 3.1.1. Response Surface

A response surface was obtained for each response variable (Table 1). The probability of disease transmission from infected to non-infected people (with or without social control measures) and the proportion of the population protected by vaccination were statistically significant for both the number of people who died or recovered from infection (*p* < 0.001). There were no observed significant differences between the models starting the simulation with 10,000 or 50,000 infected people. Therefore, the number of foci or people infected at the time of vaccination is not significant, probably due to the rapid spread of the virus in the population.

Using the model, if 40% of the population was protected by vaccination, there were 74,217 (2 × 37,108.5, Table 1) fewer estimated deaths and 9 million fewer estimated infections (20% of the total population in Spain) compared with the simulation based on 10% (low level of the experiment) of the population protected by vaccination. Moreover, if social measures were applied in the population, there were 54,666 fewer estimated deaths and 8.8 million fewer estimated infections than when no social measures were applied using the model. 

#### 3.1.2. Sensitivity Analysis

Using the surfaces obtained with the Box–Behnken design (Table 1), the sensitivity of the model to small variations in the parameters was also estimated. Small variations (1%) in the parameters (probability of disease transmission from infected to non-infected person and the percentage of people protected by vaccination) had a great impact on the outcome variables (dead or recovered people) (Table 2). 

#### 3.1.3. Vaccination Activity

The effect on the course of the pandemic due to different levels of the population protected by vaccination was compared in a population with 30,000 infected people at time 0. The vaccine was administered at time 0 and the probability of disease transmission from infected to non-infected people in the population was 0.05 or 0.1, when social measures were applied or not, respectively [30]. The population had no restrictions on normal activities, such as work, schools, or the use of restaurants and bars. 

The decrease in any outcome variable due to vaccination (Table 3) was measured by:

$$Reduction=\frac{VWV-VAVp}{VWV}\cdot 100.$$

VWV=Value obtained without vaccination

$$VAVp=Value\;when\;a\;proportion\;\left(p\right)\;of\;the\;population\;was\;vaccinated$$

**Figure 1:**
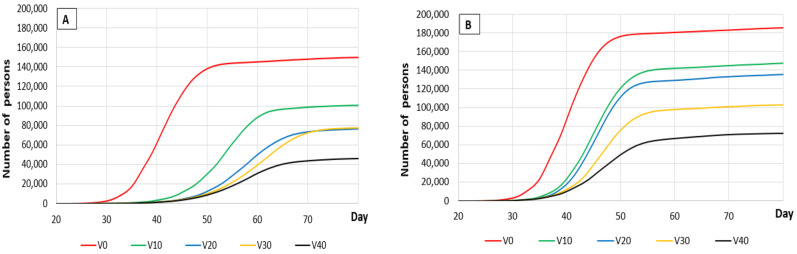

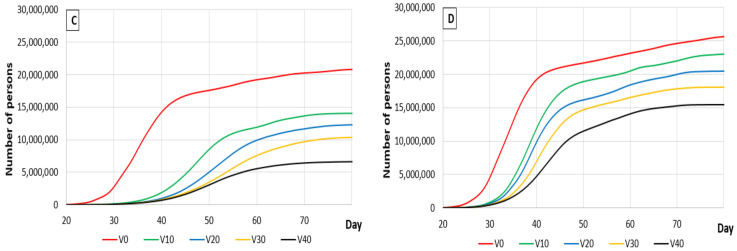
Graphs (**A**,**B**) show the progress of the death toll in Spain depending on the percentage of the population protected by vaccination (from 0% (V0) to 40% (V40)), with and without the application of social measures, respectively using a population dynamic P system (PDP) model over an 80-day period. Graphs (**C**,**D**) show the number of people recovering depending on the percentage of the population protected by vaccination (from 0% (V0) to 40% (V40)) with and without the application of social measures, respectively using a PDP model over an 80-day period.

The decreases observed, for any given probability of transmission without vaccination, were much greater when the probability of transmission was 0.05. The numbers of people who died or recovered decreased significantly as the percentage of the population protected by vaccination increased from 0% (control) to 40% (maximum level modeled, for practical reasons) (Table 3 and Figure 1). This decrease was observed independently of the probability of transmission. Moreover, as expected, reductions in the probability of transmission (when social measures were applied) contributed significantly to reducing the number of deceased and recovered people.

### 3.2. Tracing Activity

#### 3.2.1. Response Surface

Table 4 shows the results for three response variables. The number of people dying or recovering after infection, and the number of diagnostic tests to be performed, which allow quantification of the resources necessary to carry out tracing as a control measure. The probability of disease transmission and the percentage of people traced significantly affect all the response variables (*p* < 0.05). The effect of the number of initial foci in the range studied (10,000–50,000) is not statistically significant.

Following-up 40% of the positive contacts would reduce the number of people dying to 122,192 and those who eventually recover to 10,746,450, but it would be necessary to perform 40,528,888 diagnostic tests using a PDP model over an 80-day period. Reducing the probability of disease transmission from 10% (without social measures) to 5% (with social measures) plus applying contact tracing, would result in an additional reduction in the number of people dying by 34,100 and those recovering by 7,321,524; and a reduction in diagnostic tests to 10,956,670 using a PDP model over an 80-day period.

#### 3.2.2. Sensitivity Analysis

The sensitivity of the model to variations in the values of the parameters was tested using the response surfaces (Table 5). Reducing the probability of disease transmission from infected to non-infected people by 1% resulted in a reduction of 6820 deaths, 1,464,305 people recovered and more than two million diagnostic tests. An increase of 1% in the percentage of people traced reduced the numbers of deaths and recoveries by 3055 and 268,661, respectively, but increased the number of diagnostic tests required by approximately one million using a PDP model over an 80-day period.

#### 3.2.3. Effect of Tracing Measures

**Figure 2:**
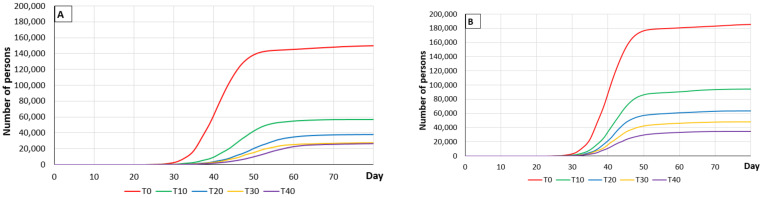

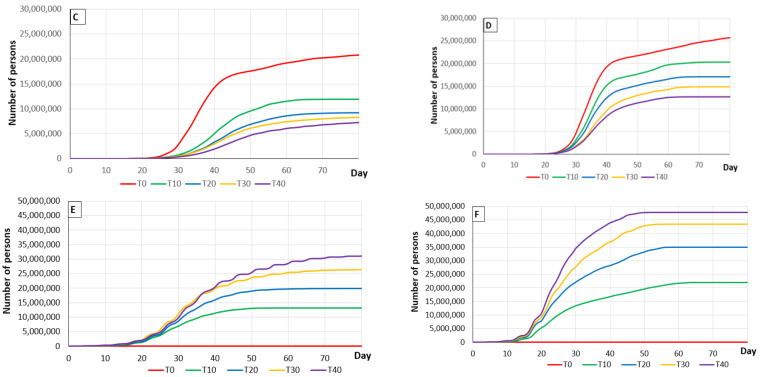
Graphs (**A**,**B**) show the progress of the death toll in Spain depending on the percentage of contacts traced (from 0% (T0) to 40% (T40)) with and without the application of social measures, respectively using a PDP model over an 80-day period. Graphs (**C**,**D**) show the number of people recovering depending on tracing contacts (from 0% (T0) to 40% (T40)) with and without the application of social measures, using a PDP model over an 80-day period. Graphs (**E**,**F**) show the number of diagnostic tests carried out depending on tracing contacts (from 0% (T0) to 40% (T40)) with and without the application of social measures, using a PDP model over an 80-day period.

The effect of contact tracing (persons in contact with someone testing positive) was studied by analyzing the percentages of the contacts. People who had been in contact with someone testing positive were monitored and quarantined in order to avoid any transmission of the disease. The effect of contact tracing 10%, 20%, 30% and 40% of the contacts was modeled and the results compared with those where contacts were not traced (Figure 2). 

In the case where social measures were applied (where the probability of disease transmission is 0.05), the results of contact tracing are significantly better (Table 6) than when social measures were not applied (where the probability of disease transmission was 0.1). Curiously, relatively small percentages of contact tracing provided important improvements in the incidence of disease cases, but this improvement did not increase linearly with the increase in the number of diagnostic tests performed. Therefore, if social measures were applied (and the probability of disease transmission was 5%), screening 10% of the positive contacts reduced the number of deaths by 0.21%, and if 40% of positive contacts were traced, the reduction was 0.27%, increasing the diagnostic tests administered from 13,106,761 to 31,767,937. Reducing the contacts traced to 20%, reduced the number of deaths by 0.25%, increasing the number of tests by 6.7 million. On the other hand, where the probability of transmission was 0.1 (without social measures), the results were similar to those where social measures were applied (with a probability of transmission of 0.05), but more diagnostic tests were carried out (Table 7). 

### 3.3. Analyzing Simultaneous Contact Tracing and Vaccine Protection

It is to be expected that only a small part of the worldwide population could be vaccinated during 2021 due to vaccine availability and logistical issues. In the case of Spain, twenty million vaccine doses, with an efficacy of around 90% for a recently authorized mRNA vaccine [26], could be available by summer 2021 [33]. This would mean that only 19.6% of the Spanish population would be protected by vaccination by the summer of 2021 (each vaccinated person requires two doses). It is estimated that at least 60–80% of the population must be immune, either through vaccination or by natural infection, to bring an end to the pandemic [34,35]. The immunity obtained by vaccination plus the immunity from natural infection would be far below this value in Spain by summer 2021. The combined effects of vaccination plus contact tracing must therefore be modeled to discover the effects of both of these disease control measures. In a final simulation setting, the percentage of the population protected by vaccination at 19.6%, the effect of tracing 10%, 20%, 30% and 40% of positive contacts was modeled in the case both of applied social measures (with 5% probability of transmission) and without social measures (with 10% probability of transmission).

**Figure 3:**
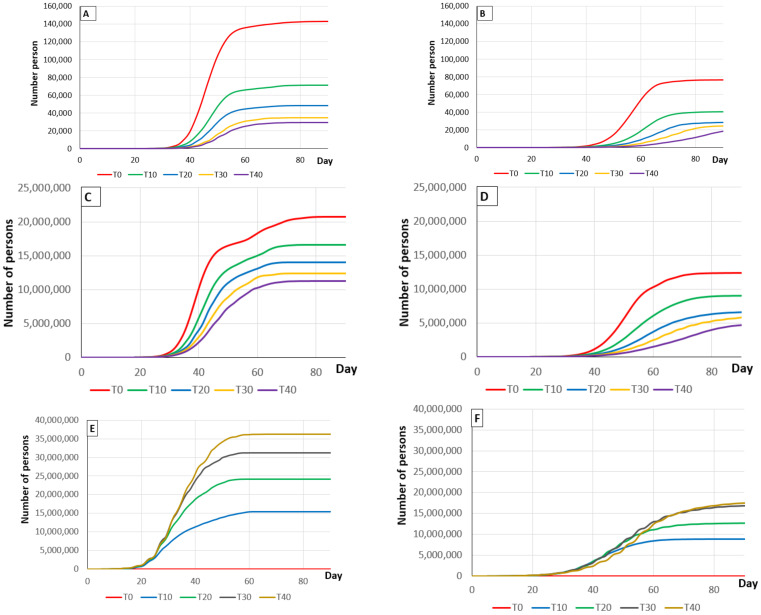
Graphs (**A**,**B**) show the progress of the death toll in Spain depending on the percentage of traced contacts (from 0% (T0) to 40% (T40)) without and with the application of social measures, with 19.6% of the population protected by vaccination, using a PDP model over an 80-day period. Graphs (**C**,**D**) show the number of people recovering depending on contact tracing (from 0% (T0) to 40% (T40)) without and with the application of social measures, with 19.6% of the population protected by vaccination using a PDP model over an 80-day period. Graphs (**E**,**F**) show the number of diagnostic tests carried out depending on contact tracing (from 0% (T0) to 40% (T40)) without and with the application of social measures, with a 19.6% of the population protected by vaccination, using a PDP model over an 80-day period.

If no social measures are applied to protect the population, it would be expected that 56.1% of the population would contract the disease and 0.4% of the population would die from it, using our PDP model over an 80-day period (Table 8 and Figure 3). Moreover, if 19.56% of the Spanish population was protected by vaccination by the end of the summer of 2021, it would be expected that 45% of the population would contract the disease and 0.3% of the population would die (Table 8 and Figure 3). However, both of these percentages are always significantly lower if social measures were adopted. The best results were obtained with social measures in place and 40% of contacts traced (Table 8 and Figure 3). Finally, if the percentage of contact tracing increases, both percentages decrease but the relationship between these decreases and the number of tests carried out declines, so that increasing the number of tests becomes relatively inefficient at tracing levels over 30% (Table 8 and Table 9).

## 4. Discussion

The strict social-distancing interventions in response to the COVID-19 pandemic during the first quarter of 2020, appear to have successfully interrupted virus transmission in many countries but this has been at the expense of huge societal disruption and economic costs [36,37]. Several modeling studies have already pointed out that resuming normal economic activities and social life would likely lead to a resurgence of the COVID-19 epidemic and this effect has been observed in many countries where restrictions were relaxed after the summer of 2020 [18,38,39]. In the light of these experiences, it is vital that policy-makers and governments identify strategies that allow economic and social activities to resume while still protecting lives and healthcare systems. To this end, mathematical models can provide new insights into the epidemiology of infectious diseases and the criteria necessary to design more efficient control strategies [40,41,42,43]. In this study, we developed models using population parameters taken as case study Spain, that is one of the most severely affected countries by the pandemic [44] but with the possibility to be applied this model on other countries only by modifying the basic parameters. 

Traditionally, ordinary differential equations (ODE), partial derivative equations and models based on stochastic processes have been used to model the dynamics of biological systems [28]. Despite their important limitations, ODEs have probably been the most widely used. They are models that deterministically describe random phenomena and use mean values without taking variability into account. In the modeling of population dynamics and epidemiological diseases there is an important randomness due mainly to the spatial distribution and movement of individuals, difficult to introduce in models based on differential equations. The estimation of the parameters in a model of differential equations is not simple in addition to being sensitive to the initial conditions. ODE models are continuous models, so there is the possibility of obtaining negative values at some point, which is not correct when modeling epidemiological diseases, nor does it make sense to give fractional results considering individuals suffering from a disease. Due to the inherent limitations of ODE models, we have developed a probabilistic model, based on PDP model methodology, to model the course of the COVID-19 epidemic under several different epidemiological scenarios. This model works with individuals (agent-based model) in which each person moves around and acts according to their own specific rules and they are grouped into bigger groups as communities in order to mimic better the dynamics of a human population. For this reason, it is extremely complicated to make direct comparison with the results obtained by other epidemiological studies due to differences in the methodology of simulation, demography and social relationship in the population, among others.

We did not consider potential changes to the virus transmissibility due to environmental factors: in particular, seasonal drivers such as temperature and humidity. Moreover, our models also did not consider possible reintroduction of SARS-CoV-2 into the population by infected travellers from other countries [45]. However, as our results suggests, the effect of the number of initial foci do not affect statistically the final result. As in any model, our results are contingent on the assumptions described, which are more thoroughly discussed by Colomer et al. (2021) [31], including the considerable uncertainties surrounding the transmission of SARS-CoV-2 which are being updated almost daily [46,47,48,49]. While our results probably do not agree exactly with reality, they do serve as useful comparisons of the likely outcomes in the presence of various control measures. For this reason, we performed extensive sensitivity analyses which show that the modeling results presented here are robust within the plausible range of parameter values for the course of the COVID-19 pandemic in Spain. Our model predicts that 56.1% and 0.41% of the Spanish population become infected or die from infection during an eighty-day period if no social measures are applied to control the disease. Fortunately, this result cannot be checked against reality because of the large number of continuing strict social control measures applied to control the disease at the beginning of the pandemic in Spain. However, our description of a baseline situation (without social control measures) is close to the values predicted in Boston, USA [39] using a different modeling approach, where 75% of the entire population became infected in a ninety-day period. Curiously, the basic R number described by these authors at epidemic peaks (R_0_ = 4) is very similar to the value used by Colomer et al., (2021) [31] in their unmitigated scenario (R_0_ = 5). On the other hand, the predicted unmitigated infection-fatality ratio was 0.63% in the UK [50], using a different modeling approach, and was higher than the value predicted in our model (0.41%). In fact, large variations in this parameter have been described in many studies, probably due to uncertainties in the key parameters chosen to run the models [51,52]. In any case, the differences that we found in the predicted numbers of people infected and dying in a baseline situation versus those described above are reasonable taking into account the different populations and models used to make these predictions that make direct comparisons between studies misleading.

Vaccination against SARS-COV-2 appears to be the most important measure to control the COVID-19 pandemic in the long run. Fortunately, several vaccines have now received approval for use as emergency measures [26,27] although it will take time before most of the population can be protected by vaccination. Our results clearly show that if 40% of the population can be vaccinated, even without social control measures, the percentage of people who die or recover from infection would fall from 0.41% and 56.1% to 0.16% and 33.5%, respectively compared with an unvaccinated population. Remarkably, if social control measures were applied in concert with vaccination the percentage of people who die or recover from infection would only be 0.10% and 14.5% after vaccinating 40% of the population. While it is clear that vaccination alone can be crucial in controlling this disease, it is necessary to vaccinate a significant part of the population and to back this up with social control measures. It must be highlighted that our model takes into account the percentage of the population protected by vaccination calculated according to the vaccine efficacy and vaccination coverage. Fortunately, the efficacies of the mRNA-based vaccines recently approved are very high (>95%) [26]. Vaccine coverage will be very similar to the percentage of the population protected by vaccination but any predictions must be revised on a case-by-case basis because other COVID-19 vaccines could have a lower efficacy than that published for mRNA-based technology [27]. Combined interventions of different degrees and intensity will therefore be necessary to substantially delay and mitigate the epidemic until herd immunity is reached by natural infection and vaccine protection [53].

Our results clearly show that social control measures are key in controlling the spread of COVID-19 in a population with a low level of vaccine protection, which will probably be the case during 2021 due to a shortage of vaccine supplies. Our findings regarding the importance of social measures to control the COVID-19 pandemic differ from earlier studies on COVID-19 during the first half of 2020 [54]. More recently, a systematic review published in December 2020 [54] suggests a significant benefit from the wearing of protective face masks in COVID-19 disease control. Recent publications have claimed that the use of face masks can reduce virus transmission by 80% [18,19]. In our model, we assumed that social measures could reduce the probability of virus transmission from infected to non-infected people by 50% because there was no information available on this matter when this study was performed. Taking into account the extra information now available, our results could be considered very conservative regarding the beneficial role of social measures in controlling the disease.

In summary, our results clearly support the extremely important role of social measures in controlling the disease at the population level [51]. On the other hand, damage has been done to society by the striking changes in people’s habits and way of life due to the long duration of the strict restrictions imposed by governments to fight the COVID-19 pandemic. The severity of the social and economic disruptions arising from social measures mean that they may not always be respected, especially in subpopulations not severely affected by the disease. Consequently, it is highly recommended that governments implement effective information campaigns on the importance of social measures. In the light of our results, it is vital that policy-makers and governments center their efforts in implementing efficient social measures and increase as much as possible contact tracing and speed up the vaccination process.

Our results clearly support the value of contact tracing as an effective tool in controlling the course of the pandemic, in common with other published studies [39,55,56] even with a low (40%), but realistic level of contact tracing. The problem with this measure is that it requires a large number of tests with a high cost for governments. It is difficult to say which of the two measures, contact tracing or social measures, is to be preferred because they require different resources. Contact tracing has financial and human resource costs while social measures require that the population conform to the measures recommended by health and government agencies. Again, our results clearly show that if everybody adheres to the prescribed social control measures, the probability of transmission is reduced and the resources required for tracing and testing fall significantly. In this regard, Li et al. 2020 [57] described how social measures have a synergistic effect with contact tracing in the control of a COVID-19 pandemic. However, strategies based on testing, isolation and contact tracing might be significantly hampered if numerous infections are imported by visitors from other areas. Travel restrictions, visitor screening and quarantine may also be required for those arriving from places where sustained local transmission rates are high [45,58,59]. In this sense, Spain is a country where the reintroductions of SARS-COV-2 viruses (with new variants) is quite probable due to international mobility on behalf of the tourism. Our model does not include this possibility because the main goal was to disentangle the effects of vaccination and contact tracing to control COVID-19 outbreak at population level where control measures (social distancing, mask wearing and hand hygiene) are or not in operation. According to our main goal, the introduction of new viruses from other countries would have added to the model an overwhelming complexity without answering the main question. This point is even more challenging for the every-day changing limitations of mobility between countries that makes the starting points in the model very speculative. Fortunately, our model is flexible enough to study this possibility in future studies.

## Figures and Tables

**Table 1:** Box–Behnken estimated values of the response surface parameters. Disease control measures, and vaccine and protection measures.

Parameter	$\mathrm{Died}\;R^{2}=0.96$		$\mathrm{Recovered}\;R^{2}=0.97$	
	Value (*)	*p*-Value	Value (*)	*p*-Value
(Intercept)	118,259.5	<0.001	17,077,712	<0.001
Probability of disease transmission (Pd)	27,333.1	<0.001	4,390,755	<0.001
% of the population protected by vaccination (V)	−37,108.5	<0.001	−4,674,854	<0.001
Number of people infected at time 0 (F)	5560.6	0.06991	−19,057	0.9136
Pd × V	−3146.7	0.41215	−318,566	0.2296
Pd × F	−1788	0.63444	64,945	0.7943
V × F	3927.8	0.31357	−13,346	0.9571
Pd^2^	−6782.2	0.1063	−915,654	0.0085
V^2^	3031	0.42859	−303,586	0.2497
F^2^	−3087.3	0.42054	331,471	0.2135

* The estimated values of the parameters are referred to the coded values, −1 and +1, of the factors (lower level and upper level).

**Table 2:** Estimated sensitivity using response surfaces. Disease control measures: vaccine and protection measures affecting the probability of disease transmission from infected to non-infected people.

**Dead** **people**	Probability of disease transmission from infected to non-infected people	10,933	People who die if the probability of transmission of the disease is increased by 1%
	Percentage of people protected by vaccination	−1855	Decrease in the number of people who died if the number of vaccinated people is increased by 1%
**Recovered** **people**	Probability of disease transmission from infected to non-infected people	1,756,302	Number of people who recovered due to a 1% increase in the probability of disease transmission.
	Percentage of people protected by vaccination	−233,743	Number of people who recovered due to a 1% increase in the population protected by vaccination.

**Table 3:** Average number of expected people who died or recovered following infection depending on the level of population protection provided by vaccination. Percentages are with respect to population size (46,014,554 people).

Social Measures		Population Protected by Vaccination (%)				
		0	10	20	30	40
**With** **(*p* = 0.05)**	Died	0.33%	0.22% (0.11%)	0.17% (0.16%)	0.17% (0.16%)	0.10% (0.23%)
	Recovered	45.34%	30.73% (14.61%)	26.93% (18.41%)	22.75% (22.59%)	14.46% (30.88%)
**Without** **(*p* = 0.1)**	Died	0.41%	0.32% (0.09%)	0.29% (0.12%)	0.22% (0.19%)	0.16% (0.25%)
	Recovered	56.11%	50.43% (5.68%)	44.55% (11.56%)	39.29% (16.82%)	33.55% (22.56%)

(Difference between the value without and with protection by vaccination).

**Table 4:** Box–Behnken estimated values of the response surface parameters. Disease control measures: positives traced and social control measures.

Parameter	Dead People $R^{2}=0.99$		Recovered People $R^{2}=0.99$		Number of Diagnostics Tests Required for Adequate Tracing $R^{2}=0.99$	
	Value (*)	*p*-Value	Value (*)	*p*-Value	Value (*)	*p*-Value
(Intercept)	48,039	<0.001	13,511,678	<0.001	27,364,890	<0.001
Probability of disease transmission (Pd)	17,049.88	<0.001	3,660,762	<0.001	5,478,335	<0.001
Percentage of people being traced (T)	−61,096	<0.001	−5,373,225	<0.001	20,264,444	<0.001
Number of people infected at time 0 (F)	433.12	0.8205	−32,519	0.8367	−92,972	0.8493
Pd × T	−14,757	0.0012	−1,025,858	0.0030	4,683,341	<0.001
Pd × F	195.25	0.9422	−365,194	0.1383	−811,020	0.2671
T × F	−78	0.9769	−17,971	0.9357	24,372	0.9719
Pd^2^	−1053.62	0.6976	−590,357	0.0327	−954,013	0.2002
T^2^	43,716.62	<0.001	2,515,438	<0.001	−6,884,809	<0.001
F^2^	2329.38	0.402	88,975	0.692	522,739	0.460

* The estimated values of the parameters are referred to the coded values, −1 and +1 of the factors (lower level and upper level).

**Table 5:** Estimated sensitivity using response surfaces. Disease control measures: tracing and social control measures.

Dead people	Probability of disease transmission from infected to non-infected people	6820	Increase in the number of people who died by increasing the probability of disease transmission by 1%
	Percentage of people being traced	−3055	Decrease in the number of people who died by increasing tracing contacts by 1%
Recovered people	Probability of disease transmission from infected to non-infected people	1,464,305	Increase in the number of people who recovered due to a 1% increase in the probability of disease transmission.
	Percentage of people being traced	−268,661	Decrease in the number of people who recovered due to a 1% increase in tracing contacts.
Diagnostic tests	Probability of disease transmission from infected to non-infected people	2,191,334	Increase in the number of tests by increasing the probability of disease transmission by 1%
	Percentage of people being traced	1,013,222	Increase in the number of tests by increasing the tracing contacts by 1%

**Table 6:** Average number of expected people who died or recovered depending on the percentage of contact tracing carried out in the population. Percentages are with respect to population size (46,014,554 people).

Social Measures		People with Positive Contacts That Have Been Traced (%)				
		0	10	20	30	40
With (*p* = 0.05)	Died	0.33	0.12 (0.21)	0.08 (0.25)	0.06 (0.27)	0.06 (0.27)
	Recovered	45.34	25.88% (19.46)	20.15 (25.19)	18.26 (27.08)	16.45 (28.89)
Without (*p* = 0.1)	Died	0.41	0.21 (0.20)	0.14 (0.27)	0.10 (0.31)	0.07 (0.34)
	Recovered	56.11	44.15 (11.96)	37.14 (18.97)	32.24 (23.87)	27.48 (28.63)

(Difference between the value without and with protection by vaccination).

**Table 7:** Number of tests performed based on the percentage of contacts traced.

Probability of Transmission of the Disease	Contacts Traced (%)	Number of People Traced	People Traced against the Total Population (%)
With social measures *p* = 0.05	0	0	0.00
	10	13,106,761	28.48
	20	19,831,667	43.10
	30	26,495,337	57.58
	40	31,767,937	69.04
Without social measures *p* = 0.1	0	0	0.00
	10	21,943,371	47.69
	20	34,913,187	75.87
	30	43,462,608	94.45
	40	47,673,047	103.60

**Table 8:** Percentage of the total number of people who died or recovered depending on the percentage of contact tracing carried out in the population if there 19.56% of the population are protected by vaccination.

Percentage of the Population		No Vaccine	19.56% of the Population Protected by Vaccination				
			Positive Contact Tracing (%)				
			0	10	20	30	40
With social measures (*p* = 0.05)	Died		0.17	0.09	0.06	0.06	0.05
	Recovered		26.96	19.60	14.46	13.26	10.62
Without social measures (*p* = 0.1)	Died	0.4	0.31	0.16	0.11	0.08	0.08
	Recovered	56.1	45.06	36.06	30.37	26.83	28.68

**Table 9:** Diagnostic tests performed as a percentage of the Spanish population (46,014,554) when vaccination protects 19.6% of the population and contact tracing follows up 10% to 40% of contacts with an infected person.

Probability of Disease Transmission	Number of Diagnostic Tests Performed with Respect to Population Size (%)			
	10% Positive Contact Tracing (%)	20% Positive Contact Tracing (%)	30% Positive Contact Tracing (%)	40% Positive Contact Tracing (%)
With social measures (*p* = 0.05)	19.3 (8,876,461)	27.7 (12,750,293)	36.8 (16,927,530)	39.0 (17,930,132)
Without social measures *p* = 0.10	33.4 (15,346,800)	52.5 (24,166,442)	68.0 (31,294,462)	78.8 (36,247,483)

## Data Availability

All the information is available from requesting to the corresponding author.

## Supplementary Materials

The following are available online at https://www.mdpi.com/article/10.3390/vaccines9040386/s1, Figure S1: Representation of a cell in a PDP, Figure S2. Example of an evolution rule, Figure S3. Process flow involved in the PDP model SARS-COV2, Table S1. Epidemiological parameters for SARS-COV2 modelling using PDP model, Table S2. Demographic parameters for SARS-COV2 modelling using PDP model.
