# Supplementary material for: Modeling of Vaccination and Contact Tracing as Tools to Control the COVID-19 Outbreak in Spain
Source: Vaccines (Basel). 2021 Apr 14;9(4):386. doi: 10.3390/vaccines9040386 (PMC8071008; doi:10.3390/vaccines9040386)
Supplement: Supplementary file 1 [file vaccines-09-00386-s001.pdf]

Supplementary Material

Modelling of vaccination and contact tracing as tools to control the COVID-19 outbreak in Spain

Mª Àngels Colomer , Antoni Margalida, Francesc Alòs, Pilar Oliva, Anna Vilella and Lorenzo Fraile

PDPs are probabilistic computational models inspired by the functioning of cells, these cells are in an environment that can be accessed by agents to evolve and move between cells (Paun et al. 2010, Colomer et al. 2013). All cells have the same hierarchical organization, which allows the generation of spaces with different and variable properties over time. The cell is represented by a rectangle (Figure S1) separated from the outside by the skin membrane. Inside the cell there are different rectangles that delimit the interior spaces. The spaces are differentiated by labels that can be numeric or alphanumeric values (subscripts). All inner membranes have electrical charge (+, -, 0) (superscript) that characterizes the cell subspace temporarily.

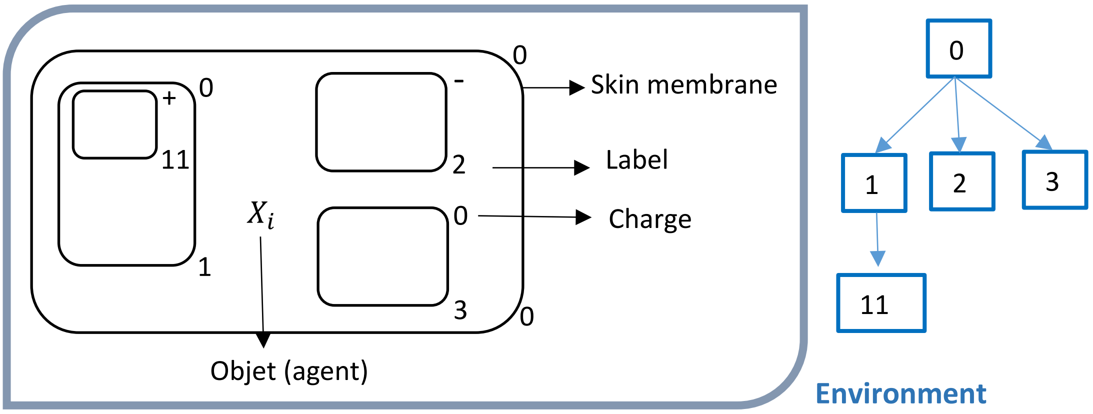

Figure S1. Representation of a cell in a PDP $[[[\ \ ]_{11}]_1[\ \ ]_2[\ \ ]_3]_0$

The agents in the case of PDP model are objects denoted by a variable that can have four subscripts to characterize it or collect information during the evolutionary process. A type of object is associated to each different input of the model and to each individual or agent an object, for example, in the case of the SARS-COV2 model, an X_i object (Figure S1) is associated to each non-infected individual in the population, the subscript indicates the age, when the individual is infected the object changes to type I_i. Not all objects are associated with people, for example d_i are objects associated with the virus that can infect a person of age i. Not all the types of objects that are going to intervene in the modeling are in the initial moment, some can be generated during the execution. Objects evolve by rules, known as rules of evolution that are expressed in a similar way to a chemical reaction (Figure S2).

$[X_i d_i]_1^- \xrightarrow{p} I_i[\quad]_1^0$<br><br>$[X_i d_i]_1^- \xrightarrow{1-p} [X_i]_1^0$	The object X_i, (individual aged i) if have contact with object d_i (have been in contact with a contagied person) with a probability p can evolve to object I_i (can be contagious) or can remains without evolving with a probability $1 - p$.<br>In order for these rules to be executed, the objects must be inside the membrane labeled 1 and also the charge on this membrane must be negative.

Figure S2. Example of an evolution rule.

The rules of evolution do nothing more than directly describes the information observed in the real problem. It is not necessary to sequence the different processes to be modeled since the model itself chooses the rules that it can execute at all times and applies them in a maximal and parallel manner

according to the existing resources at all times. The rules of evolution are included at the end of this supplementary material as Annex I.

The system is defined in a much more realistic way because it does not only take into account the average behavior, but also the dispersion of the population through the probabilities associated with the rules and the spatial distribution of the objects or agents. There is a direct correspondence between the elements in the system and the elements in the model, and the relationships between them in the system and the relationships between elements in the model.

A PDP model is defined when it is known: environments, cell membrane structure, initial objects and evolution rules (Colomer et al. 2013). Regardless of the path followed to define the model, the results that the model gives are the same. It is a top-down modeling process, in which the macroscopic properties of the systems emerge from the relationships between objects that make up the system, which makes it possible to study the systems from the point of view of the elements that make it up.

PDPs are computational models whose analysis must be done through simulations, the software that currently exists is MeCoSim.

Here, a generic model is presented that can be applied in any area divided into subzones, we specifically apply it to Spain, which is divided into 19 communities and has a total of 46 million inhabitants, so our model starts at the initial moment out of 19 environments, one per community, within each environment there are as many agents (objects) as there are inhabitants of the community, all of them have a subscript that keeps the age. At time 0, all objects are susceptible (S) to be infected, except those that are symptomatically or

asymptomatic (A) (initial outbreaks), the user of the model enters the number of infected agents from the model console and you can decide if you want the model to randomly select the agents or you can decide in which region they are and how old they are.

Starting from the initial state, the mobility and contacts that the agents have on a daily basis, the disease transmission process begins. If an agent associated with a person is in contact with an infected person, it can become infected with a probability that depends on the disease and can vary with age. The infected agent after the incubation period (variable with age) evolves, manifesting or not the disease and transmitting to new agents. It is considered that if the agent manifests the disease, it is controlled and therefore transmission is prevented.

The period of infectivity has a range of values and it is randomly generated in the model. When the infection period ends, the object evolves by recovering (R) or dying (D). (Figure S3). The recovered agents acquire an immunity that may or may not be temporary. In the case of being temporary, the average number of days it lasts is entered from the simulator console.

Parallel to the process inherent to the disease, agents can travel to other communities, the duration of the trips is another variable parameter that the user will enter, in the case of Spain it has been selected from published statistical data.

When a disease is already classified as a pandemic, governments take various management and control measures such as: reduction of mobility, space closures, monitoring of infected, massive tests and vaccines. Several of these measures can be applied simultaneously. The PDPs and specifically the PDP

SARS-COV2 allow all these measures to be totally or temporarily parallelized, selecting the time of application and duration.

In order to run the model, epidemiological parameters (Table S1) and demographic parameters (Table S2) are necessary.

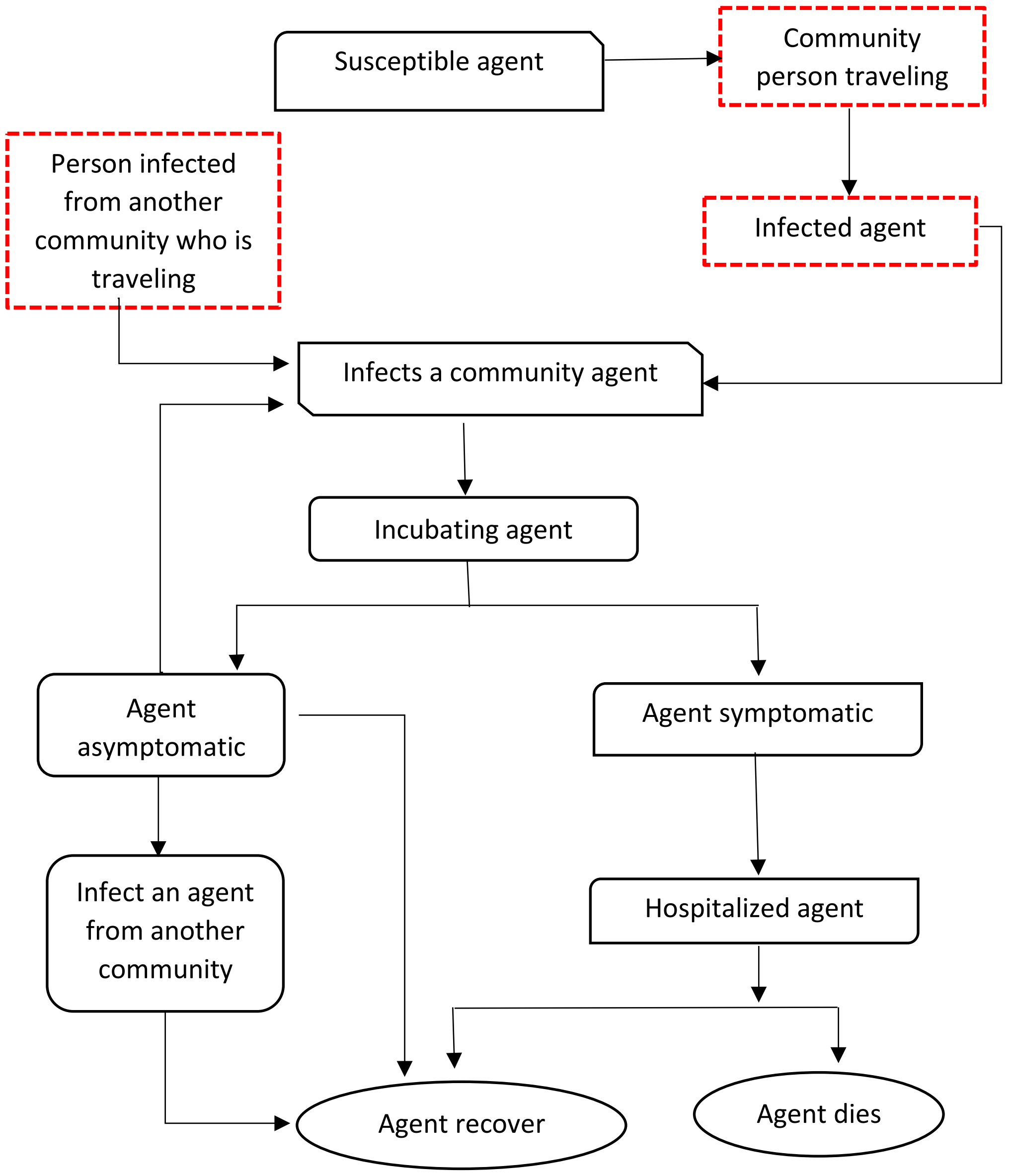

Figure S3. Process flow involved in the PDP model SARS-COV2.

Table S1. Epidemiological parameters for SARS-COV2 modelling using PDP model (see Colomer et al. under review).

**Definition parameter**	**Value**	**References**
Average duration of the virus incubation (days).	5.1± 10%	[Lauer et al. 2020, He et al. 2020, Holshue et al. 2020]
Average duration of virus transmission (days).	14± 10%	[Holshue et al. 2020, Chen et al. 2020; Hellewell et al. 2020, Anderson et al. 2020, Bi et al. 2020]
Average time needed to recover from the disease (days)	21±10%	[Lauer et al. 2020, He et al. 2020, Holshue et al. 2020]
Probability that a person, belonging to age group contacts with an infected person, becomes infected.	Variable but the same value has been taken for all age ranges in the different scenarios	
Probability that an infected person of age class will require medical treatment.	Age: Probability<br>0-2: 0.01<br>3-5: 0.01<br>6-11: 0.03<br>12-18: 0.05<br>19-25: 0.1<br>26-60: 0.15<br>61-70: 0.185	[He et al. 2020, Ferguson et al. 2020]

		70-80	0.185		
		>80	0.185		
Probability that a person of age class will die after being infected.		Age	Probability		[He et al. 2020,, Ferguson et al. 2020]
		0-2	0.003		
		3-5	0.003		
		6-11	0.003		
		12-18	0.001		
		19-25	0.003		
		26-60	0.01		
		61-70	0.044		
		70-80	0.138		
		>80	0.25		

Table S2. Demographic parameters for SARS-COV2 modelling using PDP model (see Colomer et al. under review).

**Definition**	**References**
People in the community who belong to age class	INE. Instituto Nacional de Estadística (INE), Spain, 2020. https://www.ine.es/
Number of people infected at the initial stage (required parameter if the outbreaks of infection are random)	Model option.
Number of people infected, at time 0, in the community who belong to age class (required parameter if the focuses of infection are not random).	Model option.
Number of people of age group who are in contact with people of age group applying measures of r type.<br>The model allows applying 4 types of measures	Model option.

that involve different contacts between groups of people. The case $r = 1$ corresponds to contacts assuming free movements in the population (situation previous to the pandemic), The user can define different measures that imply decreasing the number of contacts by age group. The bigger is the number, the stricter are the measures to decrease the contacts (r) $from$ 2 to 4,	
Percentage of people of age class applying the contact reduction measure r $(1 \leq r \leq 4)$.	Model option.
Probability that a person of age class travels to another community on a particular day. The same value is used (0.0005)	Model option. They have been estimated based on INE information, Spain 2020.
Average duration (days) of the trip of an age class to another community.	Model option. They have been estimated based on INE information, Spain 2020.
Probability that a person who has travelled goes from community c_1 to community c_2	Model option. They have been estimated based on INE information, Spain 2020.

References

Anderson, R.M., Heesterbeek, H., Klinkenberg, D., et al. How will country-based mitigation measures influence the course of the COVID-19 epidemic? *Lancet* **2020**, 395, 931-934.

Bi, Q., Wu, Y., Mei, S., et al. Epidemiology and transmission of COVID-19 in 391 cases and 1286 of their close contacts in Shenzhen, China: a retrospective cohort study. Lancet Infect. Dis. **2020**, 20, 911-919.

Chen, N., Zhou, M., Dong, X., et al. Epidemiological and clinical characteristics of 99 cases of 2019 novel coronavirus pneumonia in Wuhan, China: a descriptive study. *Lancet* **2020**, 395, 507-513.

Colomer, M.A., Margalida, A., Alòs, F., et al. Modelling the SARS-COV2 outbreak: assessing the useful of protective measures to reduce the pandemic at population level. Under review.

Colomer, M.À., Margalida, A., Pèrez-Jimenez, M.J. Population Dynamics P system (PDP) models: a standardized protocol for describing and applying novel bio-inspired computing tools. *PLoS One* **2013**, 8, e60698.

Ferguson, M., Laydon, D., Nedjati-Gilani, G., et al. I Report 9: Impact of non-pharmaceutical interventions (NPIs) to reduce COVID-19 mortality and healthcare demand. https://www.imperial.ac.uk/media/imperial-college/medicine/sph/ide/gida-fellowships/Imperial-College-COVID19-NPI-modelling-16-03-2020.pdf. [accessed May 30, 2020].

He, X., Lau, E.H.Y, Wu, P., et al.Temporal dynamics in viral shedding and transmissibility of COVID-19. *Nature Med.* **2020**;26, 672-675.

Hellewell, J., Abbott, S., Gimma, A., et al. Centre for the Mathematical Modelling of Infectious Diseases COVID-19 Working Group, Funk S, Eggo RM. Feasibility of controlling COVID-19 outbreaks by isolation of cases and contacts. *Lancet Glob. Health* **2020**, 8, e488-e496.

Holshue, M.L., DeBolt, C., Lindquist, S., et al. First Case of 2019 Novel Coronavirus in the United States. *N. Engl. J .Med*. **2020**, 382, 929-936.

Lauer SA, Grantz KH, Bi Q, et al. The Incubation Period of Coronavirus Disease 2019 (COVID-19) From Publicly Reported Confirmed Cases: Estimation and Application. Ann Intern Med. 2020;172(9):577-582.

Annex I

Rules of evolution of the model applied in each step (see Colomer et al. under review).

***Step 1*.** The first step is used to randomly perform PCRs on the population. They will be done on people who have not been previously diagnosed. At instant 0, there are only objects of the type X_i and INF_i, which correspond, respectively, to people who have not been infected and to people who have just been infected but who do not show the disease at the moment. When the model starts to run, different situations are generated: people who have suffered the disease asymptomatically and have already recovered, XRI_i, people who are infected asymptomatically, $I_{i,j,d}$ and people who are incubating the disease, $IC_{i,j,k,d}$. All these people are unaware of their health status so they are susceptible to realize a diagnostic test.

$r_1: INF_i, PCR[\ \ ]_2^0 \xrightarrow{\frac{1}{AINF_i+1}} IRR_{i,j,k}[\ \ ]_2^+, MNInf_i \leq k \leq MXInf_i, 1 \leq i \leq 9.$

$r_2: XRI_i, PCR[\ \ ]_2^0 \longrightarrow XRI_i[\ \ ]_2^+, 1 \leq i \leq 9.$

$r_3: X_i, PCR[\ \ ]_2^0 \longrightarrow X_i[\ \ ]_2^+, 1 \leq i \leq 9.$

$r_4: I_{i,j,d}, PCR[\ \ ]_2^0 \longrightarrow IRA_{i,j,d}[\ \ ]_2^+, 1 \leq d < j, MNInf_i \leq j \leq MXInf_i, 1 \leq i \leq 9.$

$r_5: IC_{i,j,k,d}, PCR[\ \ ]_2^0 \longrightarrow IRR_{i,k,1}[\ \ ]_2^+, 1 \leq d \leq j, MNInc_i \leq j \leq MXInc_i, MNInf_i \leq k \leq MXInf_i, 1 \leq i \leq 9.$

Counter evolution

$r_6: R_0[\ \ ]_2^0 \longrightarrow R_0[\ \ ]_2^+.$

***Step 2*.** The INF_i object is associated with a person of age i who is a carrier of the virus. Initially, the carrier will incubate the virus for a period of time and then, for another period of time, it will be able to infect other people. These times are random variables of which their mean values have been estimated with published information (Table 1 in the paper). The simulator generates random values from both time periods following normal distributions; 10 random numbers are generated for each time period and age. In this step, they are chosen randomly among the 10 of each type, which one is to be taken.

At the initial moment, there are no infected individuals from other communities, but because of the spread of the virus evolves, these cases with associated objects $INFT_{i,c}$ may appear. The first index indicates the age group to which the individual belongs and the second, the community of origin. Incubation and infection times are assigned later to these individuals. The reason is not to increase the computational cost. The fact of assigning these values later supposes a very small loss of information, considered insignificant given the low relative percentage of these objects.

In the event that not all diagnostic tests can be performed, all remaining PCR objects will be removed.

$r_9: PCR[\ \ ]_2^+ \longrightarrow [\ \ ]_2^-.$

Counter evolution.

$r_{10}: R_0[\ \ ]_2^+ \longrightarrow R_1[\ \ ]_2^-.$

Step 3. In the previous step, two numbers (from 1 to 10) have been randomly chosen, which will allow each individual to be associated with an incubation and an infection time. In this step, a time from a database is associated with each number. It is necessary to do it in two steps (r_7 and r_{11}), since when generating random numbers following a theoretical pattern, the values could be repeated and this would cause an error by not adding 1 the probabilities of evolution.

$$r_{11}: \left[INF'_{i,j,k} \longrightarrow IC_{i,floor(RandInc_{i,j}),floor(RandInf_{i,k}),1}\right]_0^0, 1 \le i \le 9, 1 \le j \le 10, 1 \le k \le 10.$$

The model allows varying the incubation and infection times depending on the age of the person. The last index of the IC object counts the specific days that this individual has been incubating the disease.

Evolution of the object associated with infected people who are traveling.

$$r_{12}: \left[INFT'_{i,c} \longrightarrow ICT_{i,c}\right]_0^0, 1 \le i \le 9, 1 \le c \le Comunity.$$

The model allows different interventions to control the disease. One is to vaccinate, in the vaccination process VV and NVV objects which are generated may be more than necessary

(in case of wanting to vaccinate more people than there are), so these surplus objects must be removed in each loop.

$r_{13}: VV[\ \ ]_1^0 \longrightarrow [\ \ ]_1^0.$

$r_{14}: NVV[\ \ ]_1^0 \longrightarrow [\ \ ]_1^0.$

Evolution of the counter: in this step the counter is used to generate OD objects, which in its subscript keeps information on the management measures taken to control the spread of the disease. The number of objects of this type generated is greater than necessary, any that are left over will be removed before the end of the loop execution.

$r_{15}: \left[R_1, Day_i \longrightarrow R_2, Day_i, OD_{D_i}^{9000000}\right]_0^0, 1 \leq i \leq Time.$

Step 4**.** In this step, all the individuals capable of spreading the disease will generate objects of the d_i type. The index indicates the age of the individual they can infect and the number of associated objects depends on the age of the person carrying the virus.

People who have finished the incubation period and they are in the period of infection but are asymptomatic can spread the disease. Symptomatic people are considered to be controlled, and therefore, they will not spread the virus. There may be some virus spread by symptomatic patients, it is accepted that it is a small value and it is disregarded. Therefore the model is going to relatively underestimate these cases. At the present time, there is no knowledge of this information. If it is relevant, it can be incorporated in the model when data is available.

Some disease control measures imposed by governments reduce the number of contacts and therefore daily infections. While these measures are not respected by the 100% of the population, there is a percentage of people who respect them ($Aply$).

$$r_{16}: \Big[IC_{i,j,k,j}, OD_r \xrightarrow{Aply_{r,i}} I_{i,k,1}, d_1^{floor(People_{i,1,r})}, d_2^{floor(People_{i,2,r})}, d_3^{floor(People_{i,3,r})}, d_4^{floor(People_{i,4,r})}, d_5^{floor(People_{i,5,r})}, d_6^{floor(People_{i,6,r})}, d_7^{floor(People_{i,7,r})}, d_8^{floor(People_{i,8,r})}, d_9^{floor(People_{i,9,r})}\Big]_0^0, \ MNinc_i \leq j \leq MXinc_i, MNinf_i \leq k \leq MXinf_i, 1 \leq i \leq 9, 1 \leq r \leq 4.$$

r_{17}: $\Big[IC_{i,j,k,j}, OD_r \xrightarrow{1-Aply_{r,i}} I_{i,k,1}, d_1^{floor(People_{i,1,1})}, d_2^{floor(People_{i,2,1})}, d_3^{floor(People_{i,3,1})}, d_4^{floor(People_{i,4,1})}, d_5^{floor(People_{i,5,1})} d_6^{floor(People_{i,6,1})}, d_7^{floor(People_{i,7,1})}, d_8^{floor(People_{i,8,1})}, d_9^{floor(People_{i,9,1})}\Big]_0^0, MNinc_i \leq j \leq MXinc_i, MNinf_i \leq k \leq MXinf_i, 1 \leq i \leq 9, 1 \leq r \leq 4.$

Asymptomatic people who are traveling to another community and can transmit the virus.

r_{18}: $\Bigg[IT_{i,c}, OD_r \xrightarrow{Aply_{r,i}} IT'_{i,c}, d_1^{floor\left(\frac{People_{i,1,r}}{g_3}+0.5\right)}, d_2^{floor\left(\frac{People_{i,2,r}}{g_3}+0.5\right)}, d_3^{floor\left(\frac{People_{i,3,r}}{g_3}+0.5\right)}, d_4^{floor\left(\frac{People_{i,4,r}}{g_3}+0.5\right)}, d_5^{floor\left(\frac{People_{i,5,r}}{g_3}+0.5\right)} d_6^{floor\left(\frac{People_{i,6,r}}{g_3}+0.5\right)}, d_7^{floor\left(\frac{People_{i,7,r}}{g_3}+0.5\right)}, d_8^{floor\left(\frac{People_{i,8,r}}{g_3}+0.5\right)}, d_9^{floor\left(\frac{People_{i,9,r}}{g_3}+0.5\right)}\Bigg]_0^0, 1 \leq c \leq Comunity, 1 \leq i \leq 9, 1 \leq r \leq 4.$

r_{19}: $\Bigg[IT_{i,c}, OD_r \xrightarrow{1-Aply_{r,i}} IT'_{i,c}, d_1^{floor\left(\frac{People_{i,1,1}}{g_3}+0.5\right)}, d_2^{floor\left(\frac{People_{i,2,1}}{g_3}+0.5\right)}, d_3^{floor\left(\frac{People_{i,3,1}}{g_3}+0.5\right)}, d_4^{floor\left(\frac{People_{i,4,1}}{g_3}+0.5\right)}, d_5^{floor\left(\frac{People_{i,5,1}}{g_3}+0.5\right)}, d_6^{floor\left(\frac{People_{i,6,1}}{g_3}+0.5\right)}, d_7^{floor\left(\frac{People_{i,7,1}}{g_3}+0.5\right)}, d_8^{floor\left(\frac{People_{i,8,1}}{g_3}+0.5\right)}, d_9^{floor\left(\frac{People_{i,9,1}}{g_3}+0.5\right)}\Bigg]_0^0, 1 \leq c \leq Comunity, 1 \leq i \leq 9, 1 \leq r \leq 4.$

In the 4 rules described above, a number of objects of the d_i type are generated, which are directly related to the number of people who are expected to be in contact with the person who carries the virus, and who are likely to become infected. The number of objects generated depends on the age group to which the virus carrier belongs.

Evolution of the counter: OI objects are generated, which in their index keep information of the type of management carried out with the closure of communities and therefore in the contact between people.

r_{20}: $R_2, Day_i[\quad]_1^0 \longrightarrow R_3, Day_i, OI_{DR_i}^{9000000}[\quad]_1^0, 1 \leq i \leq Time.$

Step 5. In this step the objects d_i are distributed proportionally to the probability of traveling or not. There is the possibility of tracking part of the positive contacts and the dr_i object will be associated with these individuals.

$$r_{21}: \left[d_i, OI_r \xrightarrow{(1-pty_{r,i})\cdot rastreig\cdot Sint_i} dr_i \right]_0^0, 1 \le i \le 9, 1 \le r \le 10.$$

$$r_{22}: \left[d_i, OI_r \xrightarrow{(1-pty_{r,i})\cdot rastreig\cdot(1-Sint_i)} dc_i \right]_0^0, 1 \le i \le 9, 1 \le r \le 10.$$

$$r_{23}: \left[d_i, OI_r \xrightarrow{(1-pty_{r,i})\cdot(1-rastreig)} dc_i \right]_0^0, 1 \le i \le 9, 1 \le r \le 10.$$

$$r_{24}: \left[d_i, OI_r \xrightarrow{pty_{r,i}} dt_i \right]_0^0, 1 \le i \le 9, 1 \le r \le 10.$$

Counter evolution.

$$r_{25}: R_3[\ ]_1^0 \longrightarrow R_4[\ ]_1^-.$$

Step 6. In this step the disease is transmitted.

A large number of objects of OD type were generated, but as they will no longer be needed, in this step those that are left over will be eliminated.

$$r_{26}: OD_i[\ ]_1^- \longrightarrow [\ ]_1^+, 1 \le i \le 4.$$

$$r_{27}: OI_i[\ ]_1^- \longrightarrow [\ ]_1^+, 1 \le i \le 10.$$

If a person who is not a carrier of the virus is in contact with a carrier, they may or may not be infected with a probability p_i.

If a carrier person is in contact with another who is incubating the disease, their health status will not change; it will be the same as if they were in contact with an asymptomatic person. The virus can be transmitted to anyone in the community, those who belong to it and those who are traveling in it.

$$r_{28}: X_i, dc_i[\ ]_1^- \xrightarrow{p_i} INF''_i[\ ]_1^+, 1 \le i \le 9.$$

$$r_{29}: X_i, dc_i[\ ]_1^- \xrightarrow{1-p_i} XS_{i,1}[\ ]_1^+, 1 \le i \le 9.$$

There are individuals who are "immune" to the disease, who are in contact with the virus but are not infected. In case a person is in contact with the virus g_5 times and are not infected, it is accepted that they are immune.

$r_{30}: XS_{i,j}, dc_i[\ \ ]_1^- \xrightarrow{1-p_i} XS_{i,j+1}[\ \ ]_1^+, 1 \leq j < g_5, 1 \leq i \leq 9.$

$r_{31}: XS_{i,j}, dc_i[\ \ ]_1^- \xrightarrow{p_i} INF''_i[\ \ ]_1^+, 1 \leq j < g_5, 1 \leq i \leq 9.$

$r_{32}: XS_{i,g_5}, dc_i[\ \ ]_1^- \longrightarrow XS_{i,g_5}[\ \ ]_1^+, 1 \leq i \leq 9.$

It is accepted that once an individual recovers, he remains immune for some time ($inmune$)

$r_{33}: XR_{i,j}, dc_i[\ \ ]_1^- \longrightarrow XR_{i,j}[\ \ ]_1^+, 1 \leq i \leq 9, 1 \leq j \leq inmune.$

Individuals who are incubating the disease and who are asymptomatic, as being in contact with the virus does not change their status.

$r_{34}: IC_{i,j,k,d}, dc_i[\ \ ]_1^- \longrightarrow IC_{i,j,k,d}[\ \ ]_1^+, 1 \leq d < j, MNinc_i \leq j \leq MXinc_i, MNinf_i \leq k \leq MXinf_i, 1 \leq i \leq 9.$

$r_{35}: I_{i,j,d}, dc_i[\ \ ]_1^- \longrightarrow I_{i,j,d}[\ \ ]_1^+, 1 \leq d \leq j, MNinf_i \leq j \leq MXinf_i, 1 \leq i \leq 9.$

Individuals who have been contacted positive and who are tracking are randomly selected

$r_{36}: X_i, dr_i[\ \ ]_1^- \xrightarrow{p_i} IRR_{i,g_{4,0}}, IG_{i,1}[\ \ ]_1^+, 1 \leq i \leq 9.$

$r_{37}: X_i, dr_i[\ \ ]_1^- \xrightarrow{1-p_i} XS_{i,1}, XG_{i,1}[\ \ ]_1^+, 1 \leq i \leq 9.$

$r_{38}: XS_{i,j}, dr_i[\ \ ]_1^- \xrightarrow{p_i} IRR_{i,g_{4,0}}, IG_{i,1}[\ \ ]_1^+, 1 \leq j < g_5, 1 \leq i \leq 9.$

$r_{39}: XS_{i,j}, dr_i[\ \ ]_1^- \xrightarrow{1-p_i} XS_{i,j+1}, XG_{i,1}[\ \ ]_1^+, 1 \leq j < g_5, 1 \leq i \leq 9.$

$r_{40}: XS_{i,g_5}, dr_i[\ \ ]_1^- \longrightarrow XS_{i,g_5}, XG_{i,1}[\ \ ]_1^+, 1 \leq i \leq 9.$

$r_{41}: IC_{i,j,k,d}, dr_i[\ \ ]_1^- \longrightarrow IRR_{i,k,d}, IG_{i,1}[\ \ ]_1^+, 1 \leq d < j, MNinc_i \leq j \leq MXinc_i, MNinf_i \leq k \leq MXinf_i, 1 \leq i \leq 9\ .$

$r_{42}: I_{i,j,d}, dr_i[\ \ ]_1^- \longrightarrow IRA_{i,j,d}, IG_{i,1}[\ \ ]_1^+, 1 \leq d \leq j, MNinf_i \leq j \leq MXinf_i, 1 \leq i \leq 9.$

Similar evolution of individuals who are traveling.

$r_{43}: XT_{i,c}, dt_i[\ \ ]_1^- \xrightarrow{p_i} INFT''_{i,c}[\ \ ]_1^+, 1 \le i \le 9, 1 \le c \le Comunity.$

$r_{44}: XT_{i,c}, dt_i[\ \ ]_1^- \xrightarrow{1-p_i} XT_{i,c}[\ \ ]_1^+, 1 \le i \le 9, 1 \le c \le Comunity.$

$r_{45}: IT'_{i,c}, dt_i[\ \ ]_1^- \longrightarrow IT'_{i,c}[\ \ ]_1^+, 1 \le i \le 9, 1 \le c \le Comunity.$

Evolution of symptomatic people. The purpose is to synchronize the model.

$r_{46}: IH_{i,j,d}[\ \ ]_1^- \longrightarrow IH'_{i,j,d}[\ \ ]_1^+, 1 \le d \le j, MNRec_i \le j \le MXRec_i, 1 \le i \le 9.$

Evolution of the rest of objects associated with vaccinated people

$r_{47}: XV_i, dc_i[\ \ ]_1^- \longrightarrow XV_i[\ \ ]_1^+, 1 \le i \le 9.$

$r_{48}: IV_i, dc_i[\ \ ]_1^- \longrightarrow IV_i[\ \ ]_1^+, 1 \le i \le 9.$

$r_{49}: IRV_i, dc_i[\ \ ]_1^- \longrightarrow IRV_i[\ \ ]_1^+, 1 \le i \le 9.$

$r_{50}: CV_i, dc_i[\ \ ]_1^- \longrightarrow CV_i[\ \ ]_1^+, 1 \le i \le 9.$

$r_{51}: XV_i, dr_i[\ \ ]_1^- \longrightarrow XV_i[\ \ ]_1^+, 1 \le i \le 9.$

$r_{52}: IV_i, dr_i[\ \ ]_1^- \longrightarrow IV_i[\ \ ]_1^+, 1 \le i \le 9.$

$r_{53}: IRV_i, dr_i[\ \ ]_1^- \longrightarrow IRV_i[\ \ ]_1^+, 1 \le i \le 9.$

$r_{54}: CV_i, dr_i[\ \ ]_1^- \longrightarrow CV_i[\ \ ]_1^+, 1 \le i \le 9.$

Counter evolution.

$r_{55}: R_4[\ \ ]_1^- \longrightarrow R_5[\ \ ]_1^+.$

Step 7. In the event that the number of contagious objects, dc_i *and* dt_i, exceeds the number of individuals of age i in any community, those that are left over are eliminated, as their function has already ended.

$r_{56}: dc_i[\ \ ]_1^+ \longrightarrow [\ \ ]_1^0, 1 \le i \le 9.$

$r_{57}: dt_i[\ \ ]_1^+ \longrightarrow [\ \ ]_1^0, 1 \le i \le 9.$

$r_{58}: dr_i[\ \ ]_1^+ \longrightarrow [\ \ ]_1^0, 1 \le i \le 9.$

After the contagion phase, a person may be symptomatic with a probability $Sint_i$. In the event of being symptomatic, this person will take on average some time to recover or die. For each individual, one of the 5 randomly generated times is chosen randomly following a normal distribution with the observed mean.

$$r_{38}: I_{i,j,1}[\ \ ]_1^+ \xrightarrow{\frac{Sint_i}{5}} IH'_{i,k,0}[\ \ ]_1^0, MNInf_i \leq j \leq MXInf_i, 1 \leq i \leq 9, 1 \leq k \leq 5.$$

$$r_{39}: I_{i,j,1}[\ \ ]_1^+ \xrightarrow{1-\ Sint_i} Iv_{i,j,1}[\ \ ]_1^0, MNInf_i \leq j \leq MXInf_i, 1 \leq i \leq 9.$$

The rest of objects associated with people evolve, so the letter v is added to the object, indicating that they are capable of traveling to another community or returning to the community of origin.

Object IRR is associated to any person who has taken a test for being a positive contact. ARI corresponds to asymptomatic and IRS to symptomatic.

$$r_{59}: IRA_{i,j,d}[\ \ ]_1^+ \longrightarrow IRA_{i,j,d+1}[\ \ ]_1^0, 1 \leq d < j, MNInf_i \leq j \leq MXInf_i, 1 \leq i \leq 9.$$

$$r_{60}: IRS_{i,j,d}[\ \ ]_1^+ \longrightarrow IRS_{i,j,d+1}[\ \ ]_1^0, 1 \leq d < j, MNInf_i \leq j \leq MXInf_i, 1 \leq i \leq 9.$$

$$r_{61}: IRR_{i,j,0}[\ \ ]_1^+ \xrightarrow{Sint_i} IRS_{i,j,1}[\ \ ]_1^0, 1 \leq d < j, MNInf_i \leq j \leq MXInf_i, 1 \leq i \leq 9.$$

$$r_{62}: IRR_{i,j,0}[\ \ ]_1^+ \xrightarrow{1-Sint_i} IRA_{i,j,1}[\ \ ]_1^0, 1 \leq d < j, MNInf_i \leq j \leq MXInf_i, 1 \leq i \leq 9.$$

$$r_{63}: IRA_{i,j,j}[\ \ ]_1^+ \longrightarrow XR_{i,1}, XRI_i[\ \ ]_1^0, MNInf_i \leq j \leq MXInf_i, 1 \leq i \leq 9.$$

$$r_{64}: IRS_{i,j,j}[\ \ ]_1^+ \xrightarrow{1-pd_i} XR_{i,1}[\ \ ]_1^0, MNInf_i \leq j \leq MXInf_i, 1 \leq i \leq 9.$$

$$r_{65}: IRS_{i,j,j}[\ \ ]_1^+ \xrightarrow{pd_i} XD_i[\ \ ]_1^0, MNInf_i \leq j \leq MXInf_i, 1 \leq i \leq 9.$$

People with symptoms are hospitalized. As a result, the objects associated with the rest evolve adding the letter, which indicates that they can travel to another community.

$$r_{66}: I_{i,j,1}[\ \ ]_1^+ \xrightarrow{Sint_i} IHH_{i,}[\ \ ]_1^0, MNInf_i \leq j \leq MXInf_i, 1 \leq i \leq 9.$$

$$r_{67}: I_{i,j,1}[\ \ ]_1^+ \xrightarrow{1-Sint_i} Iv_{i,j,1}[\ \ ]_1^0, MNInf_i \leq j \leq MXInf_i, 1 \leq i \leq 9.$$

$r_{68}: I_{i,j,d}[\ \ ]_1^+ \longrightarrow Iv_{i,j,d}[\ \ ]_1^0, 2 \le d \le j, MNInf_i \le j \le MXInf_i, 1 \le i \le 9.$

$r_{69}: IC_{i,j,k,d}[\ \ ]_1^+ \longrightarrow ICv_{i,j,k,d}[\ \ ]_1^0, 1 \le d \le j, MNInc_i \le j \le MXInc_i, MNInf_i \le k \le MXInf_i, 1 \le i \le 9.$

$r_{70}: X_i[\ \ ]_1^+ \longrightarrow Xv_i[\ \ ]_1^0, 1 \le i \le 9.$

People who are in another community and who can return to the community of origin.

$r_{71}: IT'_{i,c}[\ \ ]_1^+ \longrightarrow ITv_{i,c}[\ \ ]_1^0, 1 \le i \le 9, 1 \le c \le Comunity.$

$r_{72}: XT_{i,c}[\ \ ]_1^+ \longrightarrow XTv_{i,c}[\ \ ]_1^0, 1 \le i \le 9, 1 \le c \le Comunity.$

$r_{73}: ICT_{i,c}[\ \ ]_1^+ \longrightarrow ICTv_{i,c}[\ \ ]_1^0, 1 \le i \le 9, 1 \le c \le Comunity.$

$r_{74}: XR_{i,j}[\ \ ]_1^+ \longrightarrow XR_{i,j+1}[\ \ ]_1^0, 1 \le i \le 9, 1 \le j < inmune.$

$r_{75}: XR_{i,inmune}[\ \ ]_1^+ \xrightarrow{INM} X_i[\ \ ]_1^0, 1 \le i \le 9.$

Counter evolution

$r_{76}: R_5, Day_i[\ \ ]_1^+ \longrightarrow R_6, Day_i, OT_{DR_i}^{9000000}[\ \ ]_1^0, 1 \le i \le Time.$

The subscript of the OT_r objects stores information on whether communities are isolated on the days the measures are applied.

***<u>Step 8</u>*.** In the previous step, the objects associated with the symptomatic persons were associated with a subscript that could take values from 1 to 5. Now this number is going to be replaced by a value of the normal distribution generated randomly with a mean equal to the average time it takes to recover.

$r_{77}: \left[IHH_i \xrightarrow{1/5} IHH'_{i,k,0}\right]_0^0, 1 \le i \le 9, 1 \le k \le 5.$

The objects associated with the people who are going to travel leave the cell, going to the environment. The applied rules are those of environment that we indicate as r_e. The probability of traveling is $pty_{r,i}$, depending on age and whether government institutions have decided to restrict movement between communities.

$r_{e1}: [Xv_i, OT_r]_0^0 \xrightarrow{pty_{r,i}} Xv_i[\ \ ]_0^0, 1 \le i \le 9, 1 \le r \le 10.$

r_{78}: $\left[Xv_{i},, OT_r \xrightarrow{1-pty_{r,i}} X''_i\right]_0^0, 1 \leq i \leq 9, 1 \leq r \leq 10.$

r_{e2}: $[Iv_{i,j,d},, OT_r]_0^0 \xrightarrow{pty_{r,i}} Iv'_i, Iv_{i,j,d}[\ \ ]_0^0, 1 \leq d \leq j, MNinf_i \leq j \leq MXinf_i, 1 \leq i \leq 9, 1 \leq r \leq 10.$

r_{79}: $\left[Iv_{i,j,d},, OT_r \xrightarrow{1-pty_{r,i}} I''_{i,j,d}\right]_0^0, 1 \leq d \leq j, MNinf_i \leq j \leq MXinf_i, 1 \leq i \leq 9, 1 \leq r \leq 10.$

r_{80}: $[ICv_{i,j,k,d} \longrightarrow IC''_{i,j,k,d}]_0^0, 1 \leq d \leq j, MNinc_i \leq j \leq MXinc_i, MNinf_i \leq k \leq MXinf_i, 1 \leq i \leq 9.$

r_{e3}: $[ITv_{i,c}, OT_r]_0^0 \xrightarrow{\frac{1}{TimeT_i}\cdot(1-Return_r)} IT_{i,c}[\ \ ]_0^0, 1 \leq i \leq 9, 1 \leq c \leq Comunity, 1 \leq r \leq 10$

r_{81}: $\left[ITv_{i,c}, OT_r \xrightarrow{1-\frac{1}{TimeT_i}\cdot(1-Return_r)} IT''_{i,c}\right]_0^0, 1 \leq i \leq 9, 1 \leq c \leq Comunity, 1 \leq r \leq 10.$

r_{e4}: $[XTv_{i,c}, OT_r]_0^0 \xrightarrow{\frac{1}{TimeT_i}\cdot(1-Return_r)} XT_{i,c}[\ \ ]_0^0, 1 \leq i \leq 9, 1 \leq c \leq Comunity, 1 \leq r \leq 10.$

r_{82}: $\left[XTv_{i,c}, OT_r \xrightarrow{1-\frac{1}{TimeT_i}\cdot(1-Return_r)} XT''_{i,c}\right]_0^0, 1 \leq i \leq 9, 1 \leq c \leq Comunity, 1 \leq r \leq 10.$

r_{e5}: $[ICTv_{i,c}]_0^0 \longrightarrow ICT_{i,c}[\ \ ]_0^0, 1 \leq i \leq 9, 1 \leq c \leq Comunity.$

Counter evolution.

r_{83}: $[R_6 \longrightarrow R_7]_0^0.$

Step 9. In this step, and following the pattern of mobility between communities, the destination community is randomly chosen from those who start the trip, while those who returned go to the community to which they belong.

Objects change environment

$r_{e6}: (Xv_i)_{c1}(\ \ )_{c2} \xrightarrow{pt_{c1,c2}} (\ \ )_{c1}(Xv'_{i,c1})_{c2}, 1 \leq i \leq 9, 1 \leq c1 \leq Comunity, 1 \leq c2 \leq Comunity.$

$r_{e7}: (Iv'_i)_{c1}(\ \ )_{c2} \xrightarrow{pt_{c1,c2}} (\ \ )_{c1}(Iv''_{i,c1})_{c2}, 1 \leq i \leq 9, 1 \leq c1 \leq Comunity, 1 \leq c2 \leq Comunity.$

$r_{e8}: (XT_{i,c2})_{c1}(\ \ )_{c2} \longrightarrow (\ \ )_{c1}(X_i)_{c2}, 1 \leq i \leq 9, 1 \leq c1 \leq Comunity, 1 \leq c2 \leq Comunity, c1 \neq c2.$

$r_{e9}: (IT_{i,c2})_{c1}(\ \ )_{c2} \longrightarrow (\ \ )_{c1}(ITr_i)_{c2}, 1 \leq i \leq 9, 1 \leq c1 \leq Comunity, 1 \leq c2 \leq Comunity, c1 \neq c2.$

$r_{e10}: (ICT_{i,c2})_{c1}(\ \ )_{c2} \longrightarrow (\ \ )_{c1}(ICTr_i)_{c2}, 1 \leq i \leq 9, 1 \leq c1 \leq Comunity, 1 \leq c2 \leq Comunity, c1 \neq c2.$

Evolution of the counter: This step generates VV and NVV objects associated with the number of people who are vaccinated. The vaccination period ranges from VAC_1 to VAC_2.

$r_{84}: R_7, Day_i, C_j[\ \ ]_1^0 \xrightarrow{Vaccination} R_8, R_9, Day_i, C_j, VV^{Vpeople_j}, NVV^{NVpeople_{j,i}}[\ \ ]_1^-, 1 \leq j \leq Comunity, VAC_1 \leq i \leq VAC_2.$

$r_{85}: R_7, Day_i, C_j[\ \ ]_1^0 \xrightarrow{1-Vaccination} R_8, R_9, Day_i, C_j, NVV^{qc_j}[\ \ ]_1^-, 1 \leq j \leq Comunity, VAC_1 \leq i \leq VAC_2.$

$r_{86}: R_7, Day_i, C_j[\ \ ]_1^0 \longrightarrow R_8, R_9, Day_i, C_j, NVV^{qc_j}[\ \ ]_1^-, 1 \leq j \leq Comunity, 1 \leq i < VAC_1.$

$r_{87}: R_7, Day_i, C_j[\ \ ]_1^0 \longrightarrow R_8, R_9, Day_i, C_j, NVV^{qc_j}[\ \ ]_1^-, 1 \leq j \leq Comunity, VAC_2 < i \leq Time.$

$r_{88}: IHH'_{i,k,0}[\ \ ]_1^0 \longrightarrow IH'_{i,floor(RandRec_{i,k}),1}, [\ \ ]_1^-\ 1 \leq i \leq 9, 1 \leq k \leq 5.$

Step 10. Objects from the environment enter the cell and the initial configuration is restored to start the loop again.

$r_{e11}: Xv'_{i,c}[\ \ ]_0^0 \longrightarrow [XT_{i,c}]_0^0, 1 \le i \le 9, 1 \le c \le Comunity.$

$r_{e12}: Iv''_{i,c}[\ \ ]_0^0 \longrightarrow [IT_{i,c}]_0^0, 1 \le i \le 9, 1 \le c \le Comunity.$

$r_{e13}: X_i[\ \ ]_0^0 \longrightarrow [X_i]_0^0, 1 \le i \le 9.$

$r_{e14}: ITr_i, I_{i,j,d}[\ \ ]_0^0 \longrightarrow [I_{i,j,d+1}]_0^0, 1 \le d < j, MNInf_i \le j \le MXInf_i, 1 \le i \le 9.$

$r_{e15}: ITr_i, I_{i,j,j}[\ \ ]_0^0 \longrightarrow [XR_i]_0^0, MNInf_i \le j \le MXInf_i, 1 \le i \le 9.$

$r_{e16}: ICTr_i[\ \ ]_0^0 \longrightarrow [INF_i]_0^0, 1 \le i \le 9.$

People who are in their own community, those who have not traveled, or those who have traveled and have already reached the community of origin, are those who can be vaccinated.

$r_{89}: X''_i, VV[\ \ ]_1^- \xrightarrow{\frac{VAC_4}{100}} XV_i[\ \ ]_1^0, 1 \le i \le 9.$

$r_{90}: X''_i, VV[\ \ ]_1^- \xrightarrow{1-\frac{VAC_4}{100}} X_i[\ \ ]_1^0, 1 \le i \le 9.$

$r_{91}: X''_i, NVV[\ \ ]_1^- \longrightarrow X_i[\ \ ]_1^0, 1 \le i \le 9.$

$r_{92}: I''_{i,j,d}, VV[\ \ ]_1^- \xrightarrow{\frac{VAC_4}{100}} IV_i[\ \ ]_1^0, 1 \le d < j, MNInf_i \le j \le MXInf_i, 1 \le i \le 9.$

$r_{93}: I''_{i,j,d}, VV[\ \ ]_1^- \xrightarrow{1-\frac{VAC_4}{100}} I_{i,j,d+1}[\ \ ]_1^0, 1 \le d < j, MNInf_i \le j \le MXInf_i, 1 \le i \le 9.$

$r_{94}: I''_{i,j,d}, NVV[\ \ ]_1^- \longrightarrow I_{i,j,d+1}[\ \ ]_1^0, 1 \le d < j, MNInf_i \le j \le MXInf_i, 1 \le i \le 9.$

$r_{95}: I''_{i,j,j}, VV[\ \ ]_1^- \longrightarrow XV_i[\ \ ]_1^0, MNInf_i \le j \le MXInf_i, 1 \le i \le 9.$

$r_{96}: I''_{i,j,j}, NVV[\ \ ]_1^- \longrightarrow XRI_i[\ \ ]_1^0, MNInf_i \le j \le MXInf_i, 1 \le i \le 9.$

$r_{97}: IC''_{i,j,k,d}, VV[\ \ ]_1^- \xrightarrow{\frac{VAC_4}{100}} CV_i[\ \ ]_1^0, 1 \le d < j, MNInc_i \le j \le MXInc_i, MNInf_i \le k \le MXInf_i, 1 \le i \le 9.$

$r_{98}: IC''_{i,j,k,d}, VV[\ \ ]_1^- \xrightarrow{1-\frac{VAC_4}{100}} IC_{i,j,k,d+1}[\ \ ]_1^0, 1 \leq d < j, MNInc_i \leq j \leq MXInc_i, MNInf_i \leq k \leq MXInf_i, 1 \leq i \leq 9.$

$r_{99}: IC''_{i,j,k,d}, NVV[\ \ ]_1^- \longrightarrow IC_{i,j,k,d+1}[\ \ ]_1^0, 1 \leq d < j, MNInc_i \leq j \leq MXInc_i, MNInf_i \leq k \leq MXInf_i, 1 \leq i \leq 9.$

$r_{100}: INF''_i, VV[\ \ ]_1^- \xrightarrow{\frac{VAC_4}{100}} CV_i[\ \ ]_1^0, 1 \leq i \leq 9.$

$r_{101}: INF''_i, VV[\ \ ]_1^- \xrightarrow{1-\frac{VAC_4}{100}} INF_i[\ \ ]_1^0, 1 \leq i \leq 9.$

$r_{102}: INF''_i, NVV[\ \ ]_1^- \longrightarrow INF_i[\ \ ]_1^0, 1 \leq i \leq 9.$

$r_{103}: XR_{i,j}, VV[\ \ ]_1^- \xrightarrow{\frac{VAC_4}{100}} XV_i[\ \ ]_1^0, 1 \leq i \leq 9, 1 \leq j \leq inmune.$

$r_{104}: XR_{i,j}, VV[\ \ ]_1^- \xrightarrow{1-\frac{VAC_4}{100}} XR_{i,j}[\ \ ]_1^0, 1 \leq i \leq 9, 1 \leq j \leq inmune.$

$r_{105}: XR_{i,j}, NVV[\ \ ]_1^- \longrightarrow XR_{i,j}[\ \ ]_1^0, 1 \leq i \leq 9, 1 \leq j \leq inmune.$

Evolution of objects associated with hospitalized people.

$r_{106}: IH'_{i,j,d}[\ \ ]_1^- \longrightarrow IH_{i,j,d+1}[\ \ ]_1^0, 1 \leq d < j, MNRec_i \leq j \leq MXRec_i, ,1 \leq i \leq 9.$

$r_{107}: IH'_{i,j,j}[\ \ ]_1^- \xrightarrow{1-pd_i} XRH_i[\ \ ]_1^0, MNRec_i \leq j \leq MXRec_i, 1 \leq i \leq 9.$

$r_{108}: IH'_{i,j,j}[\ \ ]_1^- \xrightarrow{pd_i} XD_i[\ \ ]_1^0, MNRec_i \leq j \leq MXRec_i, 1 \leq i \leq 9.$

Evolution of objects associated with people who have not traveled.

$r_{109}: IT''_{i,c}[\ \ ]_1^- \longrightarrow ITvc_{i,c}[\ \ ]_1^0, 1 \leq i \leq 9, 1 \leq c \leq Comunity.$

$r_{110}: XT''_{i,c}[\ \ ]_1^- \longrightarrow XT_{i,c}[\ \ ]_1^0, 1 \leq i \leq 9, 1 \leq c \leq Comunity.$

$r_{111}: INFT''_{i,c}[\ \ ]_1^- \longrightarrow INFT_{i,c}[\ \ ]_1^0, 1 \leq i \leq 9, 1 \leq c \leq Comunity.$

$r_{112}: OT_i[\ \ ]_1^- \longrightarrow [\ \ ]_1^0, 1 \leq i \leq 10.$

The evolution of the counter must be started again. The execution of the 10-step loop results in the simulation of the events that occur throughout one day. Therefore, the

object that stores information for the day that is simulated has to increase the subscript by one unit.

$r_{113}: R_8, Day_i, C_j[\quad]_1^- \longrightarrow R_0, C_j, PCR^{Apcr_{j,i+1}}, Day_{i+1}[\quad]_1^0, 1 \leq i \leq Time - 1, 1 \leq j \leq Comunity.$

$$r_{114}: R_9[\quad]_2^- \longrightarrow [\quad]_2^0.$$
